# An Analogue of the Antibiotic Teicoplanin Prevents Flavivirus Entry In Vitro

**DOI:** 10.1371/journal.pone.0037244

**Published:** 2012-05-18

**Authors:** Tine De Burghgraeve, Suzanne J. F. Kaptein, Nilda V. Ayala-Nunez, Juan A. Mondotte, Boris Pastorino, Svetlana S. Printsevskaya, Xavier de Lamballerie, Michael Jacobs, Maria Preobrazhenskaya, Andrea V. Gamarnik, Jolanda M. Smit, Johan Neyts

**Affiliations:** 1 Rega Institute for Medical Research, K.U. Leuven, Leuven, Belgium; 2 University Medical Center Groningen, University of Groningen, Groningen, The Netherlands; 3 Fundacion Instituto Leloir, Conicet, Buenos Aires, Argentina; 4 UMR190 ‘Emergence des Pathologies Virales’, Université de la Méditerranée, Marseille, France; 5 Gause Institute of New Antibiotics, Russian Academy of Medical Sciences, Moscow, Russia; 6 Department of Medicine, Royal Free & University College Medical School, London, United Kingdom; Duke-National University of Singapore Graduate Medical School, Singapore

## Abstract

There is an urgent need for potent inhibitors of dengue virus (DENV) replication for the treatment and/or prophylaxis of infections with this virus. We here report on an aglycon analogue of the antibiotic teicoplanin (code name LCTA-949) that inhibits DENV-induced cytopathic effect (CPE) in a dose-dependent manner. Virus infection was completely inhibited at concentrations that had no adverse effect on the host cells. These findings were corroborated by quantification of viral RNA levels in culture supernatant. Antiviral activity was also observed against other flaviviruses such as the yellow fever virus and the tick-borne encephalitis virus (TBEV). In particular, potent antiviral activity was observed against TBEV. Time-of-drug-addition experiments indicated that LCTA-949 inhibits an early stage in the DENV replication cycle; however, a virucidal effect was excluded. This observation was corroborated by the fact that LCTA-949 lacks activity on DENV subgenomic replicon (that does not encode structural proteins) replication. Using a microsopy-based binding and fusion assay employing DiD-labeled viruses, it was shown that LCTA-949 targets the early stage (binding/entry) of the infection. Moreover, LCTA-949 efficiently inhibits infectivity of DENV particles pre-opsonized with antibodies, thus potentially also inhibiting antibody-dependent enhancement (ADE). In conclusion, LCTA-949 exerts *in vitro* activity against several flaviviruses and does so (as shown for DENV) by interfering with an early step in the viral replication cycle.

## Introduction

The genus flavivirus (family *Flaviviridae*) comprises several pathogens, including dengue virus (DENV), yellow fever virus (YFV), West Nile virus (WNV), tick-borne encephalitis virus (TBEV) and Japanese encephalitis virus (JEV). Flaviviruses that are pathogenic to man are transmitted to humans by bites of mosquitoes or ticks [1]. The incidence and geographical distribution of the four distinct DENV serotypes and its vector are increasing dramatically. DENV causes more than 50 million infections annually (mainly in South-East Asia and Latin America) and infections with this virus may develop into dengue hemorrhagic fever (DHF) or dengue shock syndrome (DSS) [2]–[4]. Increased disease severity has been associated with pre-existing heterologous DENV antibodies, a phenomenon described as antibody-dependent enhancement (ADE) of infection. Antibodies have been found to enhance viral entry into Fc-γ-receptor-bearing cells and to alter the antiviral immune response, leading to increased virus particle production and subsequent immune activation. During homologous re-infection, antibodies are believed to neutralize the infecting virus and provide life-long protection against disease development. Intriguingly, however, during heterologous re-infection, cross-reactive antibodies have been implicated to enhance viral replication leading to a higher infected cell mass and increased viral burden. There is neither a vaccine nor a specific antiviral therapy available [3]. This is also the case for YFV that, together with DENV, is a leading cause of hemorrhagic fever worldwide, although a highly efficacious vaccine is available [5]. Furthermore, vector-control strategies that were once successful in eliminating YFV have faltered, thereby leading to a re-emergence of the disease [5]. The World Health Organization currently estimates that there are 200,000 cases of yellow fever annually of which over 90% occur in Africa, resulting in about 30,000 deaths per year [6]. There is, as is the case for most of the other flaviviruses, no antiviral drug available for the treatment of YFV infections.

Glycopeptide antibiotics (i.e. teicoplanin, eremomycin and vancomycin) are used for the treatment of gram-positive bacterial infections. Synthetically modified glycopeptide antibiotics (SGPAs) have been reported to be endowed with *in vitro* antiviral activity against retro- and corona viruses [7], [8]. For human immunodeficiency virus (HIV) it was shown that semisynthetic glycopeptide aglycons potentially interfere with the viral entry process [9]. The mechanism by which deglycosylated SGPAs exert antiviral activity against other viruses remains unclear.

We recently demonstrated that the teicoplanin aglycon analogue LCTA-949 inhibits the replication of hepatitis C virus (HCV) by interfering with the intracellular replication of the virus [10]. We here report that LCTA-949 also exerts *in vitro* anti-flavivirus activity and does so, surprisingly, by interfering with the very early stages of the viral replication cycle.

## Results

### LCTA-949 is an *in vitro* inhibitor of flavivirus replication

**Figure 1:**
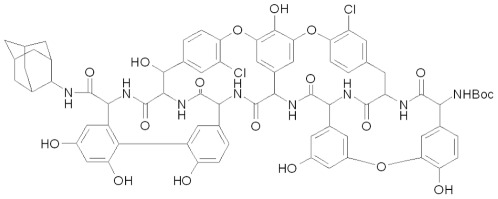
Structural formula of LCTA-949.

**Figure 2:**
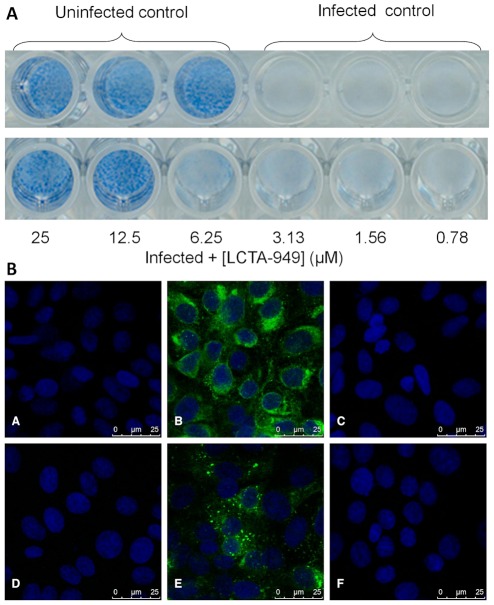
Dose-dependent inhibition of virus-induced CPE formation by LCTA-949 and effect of LCTA-949 on flavivirus protein expression. A: Vero-B cell cultures infected with DENV-2 were treated with different concentrations of LCTA-949. CPE formation was monitored at day 8 p.i. B: Vero-B cell cultures (panels A and D) were treated with 12.5 µM LCTA-949 (panels C and F) and infected with DENV-2 (panels B and C) or YFV-17D (panels E and F). DENV-2 E protein and YFV-17D NS1 protein expression was visualized on day 3 p.i.

**Figure 3:**
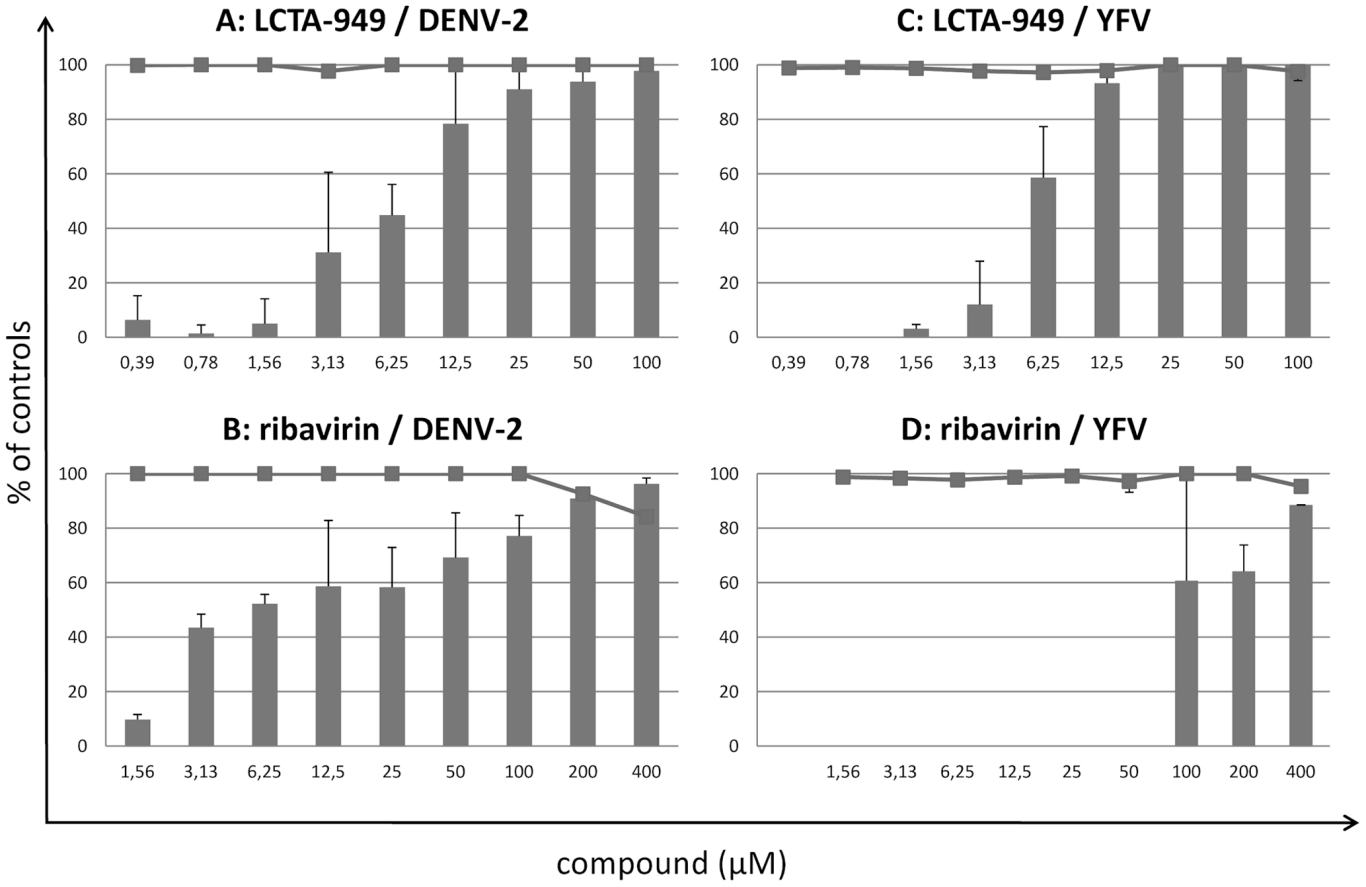
Dose-dependent inhibition of flavivirus replication by LCTA-949 and ribavirin. Vero-B cell cultures infected with DENV-2 (panels A and B) or YFV-17D (panels C and D) were treated with different concentrations of LCTA-949 (panels A and C) or ribavirin (panels B and D). Viral RNA levels were quantified on day 4 p.i. by means of RNA RT-qPCR (bars). Mock-infected cells were treated with the same dilution series of LCTA-949 or ribavirin. Cell viability was determined by the MTS/PMS method (lines). Data represent mean values ± standard deviations (SD) for three independent experiments.

The effect of LCTA-949 (Figure 1) on the *in vitro* infection of a selection of flaviviruses was evaluated in CPE-reduction assays and in virus yield reduction assays. LCTA-949 inhibits DENV-2-induced CPE formation in a dose-dependent manner (Figure 2A). At 25 and 12.5 µM LCTA-949 (concentrations that did not prove cytotoxic as assessed microscopically and by the MTS/PMS method), DENV-2-induced CPE formation was completely inhibited. Although LCTA-949 did not reduce viability of uninfected host cell cultures, at concentrations of 50 and 100 µM as assessed by the MTS method, some cytostatic effects were noted at these concentrations when cells were counted with a Coulter Counter. DENV (Figure 2B panel C) and YFV (Figure 2B panel F) protein expression (respectively E and NS1) was completely inhibited at a concentration of 12.5 µM LCTA-949. The antiviral effect of LCTA-949 was further confirmed in virus yield reduction assays [EC_50_ value of 6.9 µM±2.9 µM for DENV-2 and 5.1±3.1 µM for YFV-17D]; ribavirin was included as a reference molecule (Figure 3). In addition LCTA-949 inhibited the replication of the tick-borne encephalitis virus (EC_50_: 0.3 µM), the West Nile virus (EC_50_: 13 µM), the Japanese encephalitis virus (EC_50_: 4.3 µM), and the murine flavivirus Modoc virus (MODV) (EC_50_: 9.2 µM) (Table 1).

**Table 1:** *In vitro* antiviral effect of LCTA-949 against selected flaviviruses.

Virus	EC_50_ (µM) *	CC_50_ (µM) *	SI
DENV-2	6.9±2.9	>100	>15
YFV-17D	5.1±3.1	>100	>20
JEV	4.3±3.9	>100	>23
TBEV	0.3±0.2	>100	>333
WNV	13±4.9	>100	>7
MODV	9.2±1.2	>100	>11

* Data are mean values ± standard deviations (SD) for three independent experiments.

EC_50_: 50% effective concentration, CC_50_: 50% cytostatic concentration.

### LCTA-949 inhibits an early event in the replication cycle

**Figure 4:**
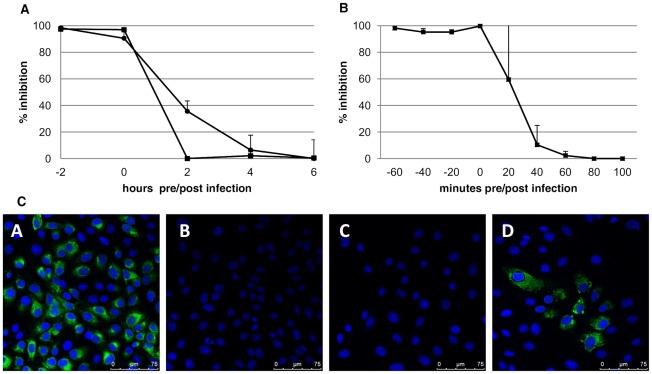
Time-of-drug-addition assay. LCTA-949 [10 µM] was added to DEN reporter virus (A, circles) or YFV-17D-infected cultures (A, squares) at 2-h intervals starting 2 hours before infection. The luciferase activity (DEN reporter virus) or the intracellular viral RNA content (YFV-17D) was measured at 24 hours p.i. Values were standardized to untreated virus controls. The data represent averages ± SD for 3 independent experiments. B: Similar conditions for YFV as in A, but time-intervals are now 20 min, starting 60 min before infection. The data represent mean values ± SD for 2 independent experiments. C: Effect of LCTA-949 on protein expression at 2-h intervals starting 2 hours before infection. DENV-2 E protein expression was visualized on day 3 p.i.

Time-of-drug-addition experiments were carried out to obtain a first indication of the stage of the viral replication cycle where LCTA-949 exerts its antiviral activity. LCTA-949 (10 µM) was added at the time of infection or at several time points before or after infection. At 24 h p.i., luciferase activity or viral antigen expression (DENV) or viral RNA content (YFV-17D) was analyzed and compared to that of untreated infected cells. Addition of the compound at the time of infection resulted in nearly complete inhibition of viral replication and viral antigen expression (Figure 4). LCTA-949 failed to efficiently inhibit DENV infection when added 2 h or later after infection (Figure 4A and 4C panel D). For YFV, most of the protective activity of LCTA-949 was already lost when LCTA-949 (10 µM) was added to the infected cultures 20 min p.i. or later (Figure 4B). Thus, LCTA-949 interferes with the earliest stages of the viral replication cycle.

### LCTA-949 does not exert a virucidal effect

To study whether the inhibitory effect of LCTA-949 is a result of direct virucidal activity, DENV-2 was incubated for 1 h (37°C) in the presence or absence of LCTA-949 (50 µM) and/or RNase A, after which viral RNA was amplified. Virus samples that had been incubated with LCTA-949 contained comparable amounts of viral RNA as samples that had not been incubated with the molecule. This demonstrates that LCTA-949 does not destroy the viral particle (data not shown). By contrast, in virus samples that had been incubated with proteinase K, viral RNA was no longer detected.

### LCTA-949 does not inhibit DENV subgenomic replicon replication

To confirm that LCTA-949 exerts its antiviral effect at the early stages of viral replication (entry/uptake) and not at a later stage, the effect of the molecule on DENV subgenomic replicon replication that encodes only non-structural viral proteins was studied. LCTA-949 did not inhibit replication of the DENV subgenomic replicon, whereas the replication inhibitor ribavirin did (data not shown). This indicates that LCTA-949 exerts its antiviral activity by targeting (one of the) structural proteins. This observation corroborates the data obtained in the time-of-drug-addition studies.

### LCTA-949 inhibits DENV entry into host cells

**Figure 5:**
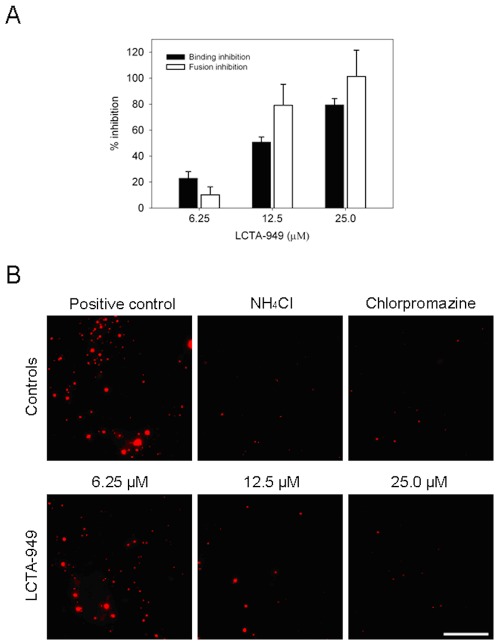
Inhibition of virus-cell binding and membrane fusion of DiD-labeled DENV by LCTA-949. BS-C-1 cells were incubated with DiD-labeled DENV in the presence or absence of different dilutions of LCTA-949 (6.25 µM, 12.5 µM and 25.0 µM). Virus cell binding was assessed after 1 h incubation at 4°C and the relative extent of membrane fusion was determined at 20 min p.i. at 37°C. (A) The percentage of binding or fusion inhibition of LCTA-949 was calculated with respect to the positive non-treated control. Ammonium chloride and chlorpromazine were used as negative controls in the fusion assay. The average of three independent experiments is shown ± SD. (B) Snapshots of the controls: no treatment, NH_4_Cl (50 mM), chlorpromazine (60 µM)] and LCTA-949 treatment are shown. Scale bar: 25 µm.

A microscopy-based binding and fusion assay using DiD-labeled virus was next employed to study whether LCTA-949 interferes with virus binding and/or entry into the host cell. DiD-labeling of DENV virions does not influence the specific infectivity of the virus [16]. The ability of DiD-labeled virions to bind to cell surface receptors at increasing concentrations of LCTA-949 was assessed in highly permissive BS-C-1 cells. In untreated cells, on average 65±15 DiD-labeled virus particles were detected. Addition of LCTA-949 markedly reduced the number of bound virus particles to cells in a dose-dependent manner (Figure 5A). At a concentration of 25 µM, LCTA-949 inhibited virus-cell binding by 80%. These results demonstrate that LCTA-949 acts by preventing virus-cell binding. Next, we investigated whether LCTA-949 has an additive effect on the membrane fusion capacity of the virus. To this end, BS-C-1 cells were infected with DiD-labeled DENV-2 particles in the presence of different concentrations of LCTA-949. The relative extent of virus fusion was estimated by measuring the total fluorescent signal per imaging field. Membrane fusion activity of DENV was efficiently reduced in cells treated with the reference controls ammonium chloride and chlorpromazine (Figure 5B) [11]. LCTA-949 markedly impaired membrane fusion activity of DENV (Figure 5A–B). This effect can likely be ascribed to the low binding efficiency of virions to cells in presence of LCTA-949, as no statistically significant added effect was measured for inhibition of membrane fusion activity as compared to the effect on binding (Figure 5A).

### LCTA-949 interferes with antibody-dependent enhancement of DENV infection

**Figure 6:**
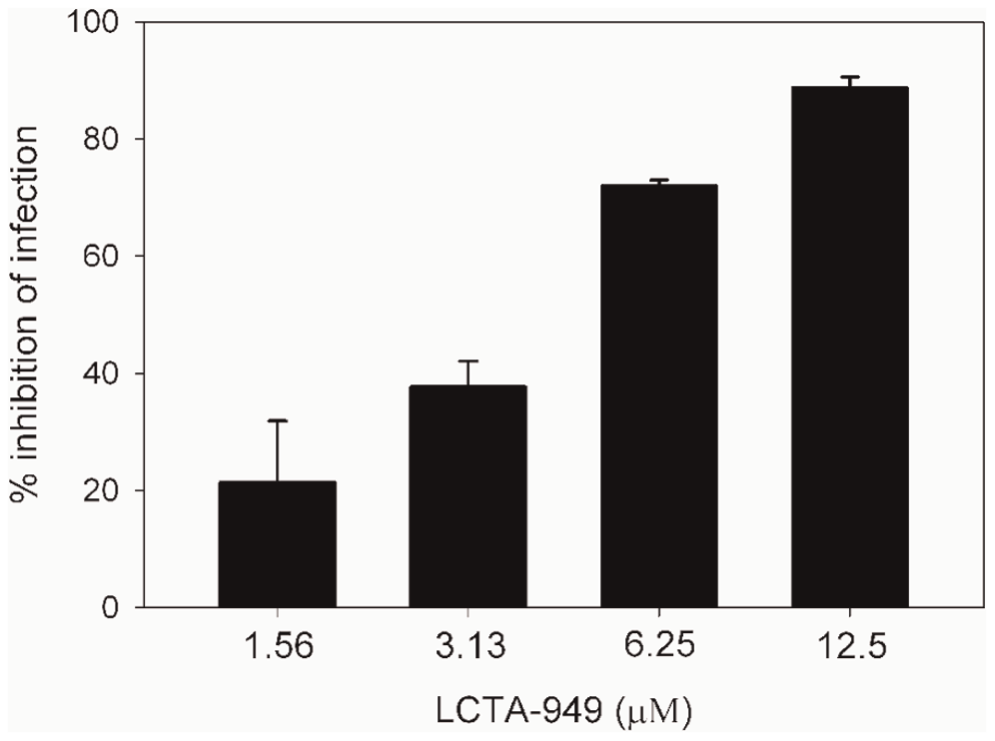
LCTA-949 prevents antibody-dependent enhancement of DENV infection. P388D1 cells were infected with DENV pre-opsonized with anti-E mAb DV2-104 at an MOI of 10 in presence of increasing concentrations of LCTA-949. Anti-E DV2-104 was used at a concentration of 400 ng/ml. The percentage of inhibition of infection after LCTA-949 treatment was calculated with respect to the positive control of untreated opsonized virus. Data are expressed as the means ± SD from two separate experiments, each of which was carried out in duplicate.

The observation that LCTA-949 prevents virus binding to the cell surface prompted us to evaluate whether LCTA-949 also exerts an antiviral effect towards DENV particles pre-opsonized with antibodies. To this end, the infectious properties of DENV particles pre-opsonized with enhancing concentrations of anti-E mAb DV2-104 was determined in the presence of increasing concentrations of LCTA-949. Under the experimental conditions, ∼0.2% of the cells were infected with DENV in the absence of mAbs, whereas in presence of enhancing concentrations of anti-E mAb DV2-104 62.8% of the cells were infected with DENV. The infectivity of anti-E DV2-104 mAb opsonized DENV particles was markedly reduced in the presence of LCTA-949 (Figure 6) [EC_50_: 4.0±0.4 µM and EC_90_: 12±1.2 µM]. To confirm that the effect observed is not antibody-specific, the effect of two other anti-E mAbs, DV2-48 or DV2-96 mapped to E domain I/II and to E domain III [12], respectively, were evaluated. In agreement with the results obtained with anti-E mAb DV2-104, the infectivity of DENV-immune complexes formed with anti-E DV2-48 and DV2-96 was significantly reduced in the presence of LCTA-949 [EC_90_s: 11±0.3 µM and 16±5 µM, respectively (data not shown)].

## Discussion

In addition to targeting viral enzymes that are indispensable for replication, interference with the virus entry step may be an attractive therapeutic strategy [13]. Flavivirus entry in the host cell consists of several dynamic events, including virus attachment, receptor-mediated endocytosis, pH-dependent membrane fusion and virus uncoating. Each of these steps can potentially serve as a target for inhibition of the virus life cycle. The DENV E protein represents an attractive target to inhibit DENV entry, since it plays a crucial role in both binding of the virus particle to host cell receptors and fusion of the viral membrane with the target membrane [14].

Polyanionic substances and plant lectins, amongst others, have been shown to be capable of inhibiting the first step of the flavivirus replication cycle, i.e. binding to the host cell. These polyanionic compounds act as heparin sulphate-mimetic substances, interfering with the interaction of the E glycoprotein with the cellular heparan sulphate receptor [14]–[17]. Some lectins, in particular those with sugar-binding specificity, were reported to prevent the interaction between the E glycoprotein and the cellular receptor DC-SIGN [18]. In addition to interfering with binding, targeting the E protein may also affect the fusion of the virus particle with cellular membranes. Virtual screening has been employed to identify molecules that interact with the β-OG pocket in the E protein [19]–[21]. Although this method seems promising, this screening still needs refinement. Additionally, we recently reported on SA-17, an analogue of the antibiotic doxorubicin, exerting anti-flavivirus activity by preventing entry of the virus, most likely via an interaction with the β-OG pocket [14].

We here report on the anti-DENV activity of LCTA-949, an aglycon derivative of the antibiotic teicoplanin. LCTA-949 inhibits not only the replication of DENV, but also that of other flaviviruses albeit with different efficiencies, including YFV, WNV, JEV and in particular TBEV. Time-of-drug-addition studies revealed that the compound exerts its antiviral activity at the time of infection and inhibits binding of the virus to the host cell. This was further supported by the observation that LCTA-949 (in contrast to the replication inhibitor ribavirin) has no effect on DENV subgenomic replicon replication and hence does not inhibit the intracellular replication machinery of the virus. Besides, a microscopy-based binding and fusion assay using DiD-labeled particles revealed that LCTA-949 inhibits DENV infection by preventing binding of the virus to the host cell. This observation may point to an interaction of LCTA-949 with the E protein; however, further studies are needed to elucidate the specific antiviral target(s) of this inhibition. A direct virucidal effect of LCTA-949 was excluded as well.

LCTA-949 was earlier identified as an inhibitor of viral infection for human immunodeficiency virus, feline and human coronavirus and hepatitis C virus [7]–[10]. The mechanism of action against HIV and coronaviruses has been ascribed to a likely effect on entry [8]. In contrast, the rather pronounced *in vitro* activity against HCV is caused by an inhibitory effect on intracellular viral replication. It is remarkable, given the fact that flaviviruses and HCV belong to the same family (*Flaviviridae*), that one and the same molecule inhibits the replication of such closely related viruses by affecting two entirely different stages in the replication cycle. An interesting example of another antiviral agent that inhibits the replication of two different viruses via a different mechanism of action (but by targeting the same cellular factor) are the cyclophilin binding molecules. Cyclosporin A and related analogues (such as the non-immunosuppressive molecules Debio-025 and MIM811) inhibit the activity of cyclophilins, which are essential co-factors in the replication of both HIV and HCV. The inhibitory effect on HIV replication is caused by an interaction with the matrix protein and thus the encapsidation process [22], whereas the effect on HCV is mediated via the cis/trans isomerisation of HCV NS5A that is required for viral RNA replication [23].

Interestingly, of all the flaviviruses studied, LCTA-949 elicited the highest antiviral activity against the TBEV (EC_50_: 0.3 µM) and proved the least active against WNV (EC_50_: 13 µM). We have as yet no explanation for the differences in responsiveness of the different flaviviruses to LCTA-949.

In order to identify the molecular target of LCTA-949, at least if this target is a viral protein, an attempt was made to generate drug-resistant variants. However, so far we have not been able to select for drug-resistant variants even not following 20 passages in the presence of suboptimal concentrations of the molecule, indicating a high barrier to resistance. We currently continue our efforts to select for drug-resistant variants. It can as yet not be excluded that LCTA-949 inhibits the entry process via interaction with a cellular target. This would mean that each of the flaviviruses that are susceptible to LCTA-949 use the same cellular factor for entry (that is targeted by LCTA-949). An interaction with the host cell membrane that disturbs the flavivirus entry process can also not been excluded. However, if this should be the mechanism, it may be difficult to explain that the entry process of the closely related HCV is not affected by this molecule. Altogether, the precise mechanism by which LCTA-949 inhibits the binding/entry process remains to be elucidated.

We found that LCTA-949 also interferes with antibody-mediated cell entry of DENV particles. ADE is thought to play a pivotal role in the exacerbation of DENV-induced disease during a secondary infection (DHF or DSS). Hence, drugs with properties like those of LCTA-949 could be of particular interest for intervention strategies. LCTA-949 was added to preformed virus-antibody complexes and was also shown to result under these conditions in an antiviral effect. These results suggest that LCTA-949 does not interfere with DENV infection through direct binding to the virus receptor since antibody-mediated cell entry of DENV is facilitated by Fc-receptors and not by the virus receptor. Indeed, these results strengthen the hypothesis that LCTA-949 binds not by the host cell receptor of the virus. However, the inhibitory effect is exerted very early since the molecule was washed away in the ADE experiments 1 hr after infection. The particular mechanism by which LCTA-949 inhibits ADE remains to be elucidated.

In conclusion, we here report on a teicoplanin aglycon analogue that exerts broad-spectrum activity against flaviviruses *in vitro* and that interferes with an early step in the viral replication cycle (binding/entry) including antibody dependent enhancement of DENV infection. Insights in the precise mechanism by which LCTA-949 exerts its antiviral activity may allow to rationally designing more potent and selective inhibitors of flavivirus entry.

## Materials and Methods

### Cells and viruses

DENV serotype 2 New Guinea C [DENV-2 NGC (kindly provided by Dr. V. Deubel (formerly at Institute Pasteur, Paris, France)] were cultured on C6/36 mosquito cells (from *Aedes albopictus*; American Type Culture Collection (ATCC) CCL-1660) in Dulbecco's modified Eagle's medium (DMEM; Gibco, Belgium) with 1% L-glutamine (Gibco), 1% penicillin (100 U/ml)/streptomycin (100 µg/ml) solution (Gibco), 1% non essential amino acids (Gibco), 1% hepes and 8% foetal bovine serum (FBS; Integro, The Netherlands) at 28°C. DENV-2 strain 16681, kindly provided by Dr. Claire Huang (Center for Disease Control and Prevention, USA), was propagated in C6/36 cells as described before [24]. BS-C-1 cells (African Green Monkey kidney cells) were maintained at 37°C and 5% CO_2_ in 1× high glucose, L-glutamine-enriched DMEM (PAA) with 10% FBS, penicillin (100 U/mL), and streptomycin (100 µg/mL). Mouse macrophage P388D1 cells were maintained in DMEM supplemented with 10% FBS, penicillin (100 U/ml), and streptomycin (100 µg/ml), sodium bicarbonate (Invitrogen, 7,5% solution) and 1.0 mM sodium pyruvate (Gibco) at 37°C, 5% CO_2_. Green monkey kidney cells [Vero-B cells (ECACC for DENV assays and ATCC CCL-81 for YFV assays)] were grown in minimum essential medium MEM Rega-3 (Gibco) supplemented with 10% FBS, 1% L-glutamine and 1% sodium bicarbonate (Gibco). Antiviral assays were performed in medium with 2% FBS. Baby hamster kidney cells (BHK-21; ATCC CCL-10) were grown in DMEM supplemented with 10% FBS (culture medium) or 2% FBS (assay medium). BHK-21 cells harboring the subgenomic dengue replicon dCprMEPAC2NS3lucNS3 (derived from the dengue replicon construct pDENΔCprME-PAC2A) in which an antibiotic selection cassette encoding the puromycin *N*-acetyltransferase (PAC) together with the *Firefly* luciferase expression cassette was inserted upstream of the non-structural (NS) genes, will be referred to as BHK-Rep-Pac-LUC cells [25]. BHK-Rep-Pac-LUC cells were cultured as the parental BHK-21 cells with the exception that 3.3 µg/ml of puromycin was added to the culture medium (Sigma-Aldrich, Belgium). Puromycin was omitted from the culture medium in antiviral assays. The construction of an infectious, full-length dengue virus (DENV-2 16681), in which a *Renilla* luciferase expression cassette under the translational control of the encephalomyocarditis virus internal ribosome entry site (EMCV IRES) was inserted upstream of the 3′ untranslated (3′UTR) region, was reported earlier [26]. Here this virus is referred to as “dengue reporter virus”. Yellow fever virus (YFV) 17D vaccine strain (Stamaril®) [Aventis Pasteur (MSD, Belgium)] was passaged once in Vero-B cells to prepare a working virus stock and stored at −80°C until further use. Modoc virus (MODV) strain M544 (ATCC VR415) was propagated in BHK-21 cells. All work using infectious JEV strain SA-14, TBEV strain Oshima and WNV strain NY99 was carried out in a biosafety level 3 laboratory. The batches of the viral inoculum (JEV, TBEV and WNV) were prepared by culturing virus twice on Vero cells in MEM supplemented with 7% FBS, 2 mM L-glutamine and penicillin-streptomycin. Finally, cell culture supernatants were collected and frozen at −80°C in 50 mM HEPES.

### Antiviral molecules

The teicoplanin analogue LCTA-949 (Figure 1) was synthesized as reported elsewhere [9]. Ribavirin [1-(β-D-ribofuranosyl)-1H-1,2,4-triazole-3-carboxamide (Virazole; RBV)] was purchased from ICN Pharmaceuticals (Costa Mesa, CA).

### CPE-reduction assay

Vero-B cells were seeded in 96-well plates (Becton Dikinson Labware, Franklin Lakes, NJ) at a density of 7×10^3^ cells/well in 100 µl assay medium and were allowed to adhere overnight. Subsequently, a compound dilution series was added after which cultures were infected with 100 CCID_50_ (i.e., 50% cell culture infectious dose) DENV-2 NGC in 100 µl assay medium. Plates were incubated at 37°C [95–99% relative humidity and 5% CO_2_]. On day 8 post infection (p.i) the cultures were fixed with 70% ethanol and stained with 1% methylene blue. Ribavirin was included in the assay as a reference compound.

### Virus yield reduction assay

Vero-B cells (5×10^4^) were seeded in 96-well plates. One day later, culture medium was replaced with 100 µl of assay medium containing a 2× serial dilution of the compound and 100 µl of virus inoculum [either DENV-2 (NGC), YFV-17D or MODV; 50 CCID_50_/well]. Following a 2 hour incubation period, the cell monolayer was washed 3 times with assay medium to remove non-adsorbed virus and cultures were further incubated for 4 days in the presence of the inhibitor. Supernatant was harvested and viral RNA load was determined by real-time quantitative RT-PCR. The 50% effective concentration (EC_50_), which is defined as the compound concentration that is required to inhibit viral RNA replication by 50%, was determined using logarithmic interpolation. Ribavirin was included as a reference compound. Antiviral assays for JEV, TBEV and WNV were carried out in Vero-B cells in 24-well plates (5×10^4^ cells/well). Briefly, cells were cultured in MEM supplemented with 7% FBS, 2 mM L-glutamine, and penicillin-streptomycin. One day after seeding, cells were infected with 100 µl of the viral inoculum (at a multiplicity of infection of either 0.1 or 1) in the presence or absence of LCTA-949. Following incubation for 90 min (37°C; 5% CO_2_), cultures were washed twice with Hanks balanced salt solution (HBSS) after which 1 ml fresh medium either supplemented or not with the compound was added to the wells. Cells were further incubated at 37°C (medium with compound was refreshed at days 3 and 5 p.i). At various time points p.i., supernatants were harvested and stored at −80°C for later quantifications by means of RT-PCR. Potential cytotoxic/cytostatic effects of the compound were evaluated in uninfected cells by means of MTS/PMS method as described earlier [27]. The 50% cytotoxic concentration (CC_50_; i.e., the concentration that reduces the total cell number by 50%) was calculated using logarithmic interpolation.

### Quantitative reverse transcriptase-PCR (qRT-PCR)

RNA was isolated from 150 µl supernatant with the NucleoSpin RNA virus kit (Macherey-Nagel, Germany) as described by the manufacturer. Primers and probe sequences are described earlier [27]. The TaqMan probe was fluorescently labeled with 6-carboxyfluorescein (FAM) at the 5′ end as the reporter dye and with minor groove binder (MGB) at the 3′ end as the quencher. One-step, quantitative RT-PCR was performed in a total volume of 25 µl, containing 13.9375 µl H_2_O, 6.25 µl master mix (Eurogentec, Belgium), 0.375 µl forward primer, 0.375 µl reverse primer, 1 µl probe, 0.0625 µl reverse transcriptase (Eurogentec) and 3 µl sample. RT-PCR was performed using the ABI 7500 Fast Real-Time PCR System (Applied Biosystems, Branchburg, NJ) using the following conditions: 30 min at 48°C and 10 min at 95°C, followed by 40 cycles of 15 s at 95°C and 1 min at 60°C. The data was analyzed using the ABI PRISM 7500 SDS software (version 1.3.1; Applied Biosystems). For absolute quantification, standard curves were generated using 10-fold dilutions of template preparations of known concentrations.

### Immunofluorescence assay

Vero-B cells were seeded in a 8-well chamber slide (Lab-tek, II, Nunc, Germany) at a density of 2×10^4^ cells/well; 24 hours later, cells were infected with 50 CCID_50_ DENV-2 NGC or YFV-17D in the presence or absence of 12.5 µM LCTA-949. The virus inoculum was removed after 1 hour; cells were washed and further incubated in the presence of the compound for 72 hours. Cells were stained with the anti-dengue E protein antibody (Ab) clone 3H5 (Millipore, Billerica, MA) or the anti-YFV NS1 Ab 1A5, and the secondary Ab Alexa Fluor 488 (Millipore) [28]. Following DAPI staining, the cultures were visualized using a confocal laser scanning microscope (LCSM, Leica Microsystems, Germany).

### Time-of-drug-addition assay

One day prior to infection, 2×10^4^ BHK-21 cells were seeded in a tissue culture-treated white view 96-well plate (Perkin Elmer, Boston, M.A). The next day, cells were infected with 2×10^5^ PFU/well of the dengue reporter virus. After one hour, the virus inoculum was replaced by assay medium. LCTA-949 at a concentration of 10 µM was added to the assay medium 2 hours before infection, or at 0 (i.e., together with the virus), 2, 4 and 6 hours post infection. Luciferase activity was measured at 24 hours p.i. using the *Renilla* Luciferase Assay System (Promega, The Netherlands). Luciferase activity of treated, infected cells was compared to that of untreated, infected cells. For experiments with YFV, confluent Vero-B cells [in a 12-well plate (Iwaki, Asahi Techno Glass, Japan)] were treated with 10 µM LCTA-949 and infected with 50 CCID_50_ of YFV in a similar way as was done for DENV. At 24 hours p.i. the intracellular RNA was isolated using the RNeasy minikit (Qiagen) and YFV-17D RNA levels were quantified using RT-qPCR. In addition, a more elaborate assay was performed for YFV in which the compound was added to the cell cultures every 20 minutes beginning 60 minutes before infection up until 100 minutes after infection.

Vero-B cells were seeded in a 8-well chamber slide (Nunc) at a density of 2×10^4^ cells/well. The next day, the cells were infected with 50 CCID_50_ DENV-2 NGC. One hour later, the inoculum was replaced by assay medium. LCTA-949 at a concentration of 10 µM was added to the assay medium 2 hours before infection, together with the virus or 2 hours post infection. Cells were stained as described before.

### DENV Replicon system

BHK-Rep-Pac-LUC cells were seeded at a density of 1×10^4^ cells/well in a tissue culture-treated white view 96-well plate (Perkin-Elmer). The next day, medium was replaced by a two-fold serial dilution of LCTA-949. After 72 hours, luciferase activity was measured using the Luciferase Assay System according to the manufacturer's protocol (Promega). Luciferase activity was compared to that of untreated replicon cells. The cytotoxic effect of the compounds on BHK-Rep-Pac-LUC cells was evaluated in parallel cultures by using the MTS/PMS assay [27]. Ribavirin was included, for comparative reasons, as a replication inhibitor. The inhibitory effect of the compounds on luciferase activity was adjusted for inhibitory effects on cell proliferation.

### Virucidal assay

An undiluted stock of DENV-2 (∼1×10^6^ PFU, 50 µl) was incubated at 37°C in the presence or absence of 50 µM LCTA-949 and in the presence or absence of 80 µg/ml RNase A (Promega). After one hour the RNase A was inactivated with 1 mg/ml proteinase K (Promega). Viral RNA was isolated using the Nucleospin kit (Filter Services) and the samples were subjected to RT-PCR and gel electrophoresis. The positive control consisted of virus incubated with 1 mg/ml proteinase K; the enzyme was inactivated after 1 hour with 5 mM phenylmethanesulfonyl fluoride (PMSF; Sigma-Aldrich) for 15 minutes and RNase A was added. Following 1 hour of incubation, RNA was isolated from the samples and analyzed.

### DiD-labeling of DENV particles

The lipophilic fluorescent probe 1,1′-dioctadecyl-3,3,3′,3′-tetramethylindodicarbocyanine, 4-chlorobenzenesulfonate salt (DiD) (Molecular Probes, Eugene, OR) was used to label the viral membrane of DENV-2 virus particles, as previously described [29]. In brief, DENV-2 16681 wild type virus was propagated in C6/36 cells and harvested at 3 days p.i. Subsequently, the virus was purified on a potassium-tartrate density gradient (10 to 35%, wt/vol) by ultracentrifugation (Beckman type SW41 rotor, 2 h, 125,000× g, 4°C) and stored at −80°C. Virus preparations were analyzed with respect to the infectious titer (PFUs) and the number of genome-containing particles (GCPs), as described before [29]. Before labeling, tartrate was cleared by using a 100 kDa filter device (Millipore, The Netherlands). While vortexing, approximately 10^10^ GCPs of virus (equivalent to ∼10^8^ PFUs) was mixed with 2 nmol DiD dissolved in dimethyl sulfoxide (DMSO) with an end concentration of DMSO of ∼2.0%. To remove unbound dye, the DiD-labeled virus was filtered with a Sephadex G-50 Fine (Pharmacia, Uppsala, Sweden) column prepared in a glass pasteur pipette. Different fractions were collected, stored at 4°C and used within 2 days.

### Binding and fusion assays

BS-C-1 cells were seeded in a Lab-Tek II Chambered Coverglass (Nunc No. 155409) such that the well was 50 to 70% confluent at the day of the infection. Prior to the binding assay, cells were incubated for 10 min at 4°C. Subsequently, DiD-labeled DENV was added in the presence or absence of LCTA-949 or a DMSO control, and incubation was continued for 1 h at 4°C. Unbound virus was removed by gently washing the cells three times with cold phenol red-free MEM. Subsequently, cold phenol red-free MEM containing 1% glucose and a glucose oxidase solution (GLOX) was added to the cells and the microscopic analysis was done as described below. GLOX is composed of a 250 µM of glucose oxidase (Type VII from *Aspergillus niger*, Sigma Aldrich No. G2133) solution, prepared in 200 µL of 1× PBS, and 50 µL of catalase (from beef liver, Roche No. 10106810001). For the fusion assay, the same experimental setup was used except that the cells were kept at 37°C during the experiment. Ammonium chloride (NH_4_Cl, 50 mM) and chlorpromazine (60 µM) were used as negative controls in the fusion assay. The microscopy analysis was done by taking 10 snapshots of randomly selected fields using both differential interference contrast (DIC) and DiD channels. DIC images were taken from each field to make sure that the visualized DiD-particles are bound to a cell and not lying in an empty space with no attached cells. DiD-labeled viruses were detected by epifluorescence microscopy in a Leica Biosystems 6000B instrument by using a 635-nm helium–neon laser. The fluorescent emission was collected by an oil-immersion 100× objective with a numerical aperture of 1.46 (Leica Microsystems) and imaged using an EM CCD camera (Hamamatsu 9100-02). A thermostatted stage and objective heater were used to keep the temperature at 37°C in the fusion assay. The acquired images were processed and analyzed with ImageJ using an in-house macro. In the binding assay, the total number of bound DiD-particles was counted. The extent of membrane fusion was analyzed by measuring the total fluorescent signal per imaging field at 20 minutes post infection. The quantitative results of the negative controls in the fusion assay were taken as background; hence, they were subtracted from the total fluorescence result of the positive control and LCTA-949 treatments.

### ADE-inhibition assay

Virus or virus-antibody complexes were added to 2×10^5^ P388D1 cells at MOI 10 of DENV-2 16681 in the presence or absence of serial two-fold dilutions of LCTA-949, and incubated at 37°C with 5% CO_2_ for 1 h. DENV-2 anti-E antibodies were generously provided by M. Diamond (Washington University, St. Louis, USA). Cells were then washed with PBS and new media was added. At 43 hpi, the cells were fixed and stained for FACS analysis as previously described [30]. The number of infected cells was determined using the anti-E monoclonal antibody MAB8702 (Millipore). For virus-antibody complex formation, virus particles were incubated for 1 h at 37°C with murine anti-E antibodies, DV2-104 (400 ng/ml), DV2-48 or DV2-96 in cell culture medium containing 2% FBS prior to the addition to cells [31]. The EC_50_ and EC_90_ were defined according to the percentage of infectivity inhibition relative to that of the positive control. No cytotoxic effects of LCTA-949 were observed at the concentrations evaluated.
